# Acquisition and Extinction of Human Avoidance Behavior: Attenuating Effect of Safety Signals and Associations with Anxiety Vulnerabilities

**DOI:** 10.3389/fnbeh.2014.00323

**Published:** 2014-09-15

**Authors:** Jony Sheynin, Kevin D. Beck, Richard J. Servatius, Catherine E. Myers

**Affiliations:** ^1^Department of Veterans Affairs, New Jersey Health Care System, East Orange, NJ, USA; ^2^Joint Biomedical Engineering Program, New Jersey Institute of Technology and Graduate School of Biomedical Sciences, Rutgers, The State University of New Jersey, Newark, NJ, USA; ^3^Stress and Motivated Behavior Institute, Department of Neurology and Neurosciences, New Jersey Medical School, Rutgers, The State University of New Jersey, Newark, NJ, USA

**Keywords:** avoidance, anxiety disorders, anxiety vulnerability, safety signal, individual differences, sex differences, inhibited temperament, computer-based task

## Abstract

While avoidance behavior is often an adaptive strategy, exaggerated avoidance can be detrimental and result in the development of psychopathologies, such as anxiety disorders. A large animal literature shows that the acquisition and extinction of avoidance behavior in rodents depends on individual differences (e.g., sex, strain) and might be modulated by the presence of environmental cues. However, there is a dearth of such reports in human literature, mainly due to the lack of adequate experimental paradigms. In the current study, we employed a computer-based task, where participants control a spaceship and attempt to gain points by shooting an enemy spaceship that appears on the screen. Warning signals predict on-screen aversive events; the participants can learn a protective response to escape or avoid these events. This task has been recently used to reveal facilitated acquisition of avoidance behavior in individuals with anxiety vulnerability due to female sex or inhibited personality. Here, we extended the task to include an extinction phase, and tested the effect of signals that appeared during “safe” periods. Healthy young adults (*n* = 122) were randomly assigned to a testing condition with or without such signals. Results showed that the addition of safety signals during the acquisition phase impaired acquisition (in females) and facilitated extinction of the avoidance behavior. We also replicated our recent finding of an association between female sex and longer avoidance duration and further showed that females continued to demonstrate more avoidance behavior even on extinction trials when the aversive events no longer occurred. This study is the first to show sex differences on the acquisition and extinction of human avoidance behavior and to demonstrate the role of safety signals in such behavior, highlighting the potential relevance of safety signals for cognitive therapies that focus on extinction learning to treat anxiety symptoms.

## Introduction

Avoidance behavior is the performance or the withholding of a specific response to prevent an upcoming aversive event (active or passive avoidance, respectively). Although normally an adaptive behavior that protects one from harm, avoidance can be over-expressed and become pathological. Indeed, exaggerated avoidance behavior is a predominant symptom in all anxiety disorders (e.g., American Psychiatric Association, 2000) and its severity often parallels the overall growth and persistence of the disorders (Karamustafalioglu et al., 2006). Much of our current understanding of avoidance behavior is based on animal literature. A common approach to assess avoidance in animals is to expose a rodent to an aversive event (e.g., electric shock), which is preceded by a warning signal (e.g., tone) and which can be avoided by performing or withholding a specific operant response (e.g., lever-press and step-down on an electrified grid, respectively). Responding (or withholding the response) during the aversive event represents an escape response (ER) that terminates the aversive event, whereas responding during the warning signal completely prevents the aversive event and thus represents an avoidance response (AR).

Avoidance behavior in rodents has been shown to depend on individual differences. The strain (Sutterer et al., 1980; Bond, 1981; Kuribara, 1982; Berger and Starzec, 1988; Servatius et al., 2008) and sex (Beatty and Beatty, 1970; Scouten et al., 1975; Van Oyen et al., 1981; Heinsbroek et al., 1983; Beck et al., 2010) of the tested animals affect the rate and overall level of active avoidance behavior acquisition in rodents. In addition, features of the protocols, such as the interstimulus interval duration (Berger and Brush, 1975) and the properties of the aversive event (D’Amato and Fazzaro, 1966) can also influence active avoidance learning. In some cases, individual differences in active avoidance learning can interact with differences in the avoidance training protocol (e.g., Beck et al., 2011). These findings suggest susceptibility to acquire avoidant behavior is not uniform; instead susceptibility is determined by sensitivity to specific stimuli or reactions to stimuli experienced during training.

Safety periods, i.e., periods free from aversive events, represent an appetitive component of avoidance behavior (Denny and Weisman, 1964), and can also modulate avoidance behavior in rodents (Berger and Brush, 1975). It was argued that signals associated with safety periods [i.e., safety signals (SSs)] provide positive reinforcement for an AR (Seligman and Johnston, 1973; Rachman, 1984) and may become inhibitors of fear (Falls and Davis, 1997; Myers and Davis, 2004). Research that examined the effect SSs on avoidance behavior showed that by introducing a visual SS during the intertrial period, acquisition of ARs was facilitated (Bower et al., 1965; Dillow et al., 1972; Hurwitz et al., 1972; Candido et al., 1991). It has been argued that the facilitation was the result of the feedback stimulus, contingent on the animal’s AR (Bolles and Grossen, 1969; Dillow et al., 1972). In agreement with this idea, when a non-contingent SS was used, no facilitation was shown (Fernando et al., 2013). Interestingly, the length of the SS did not affect acquisition of avoidance responding (Galvani and Twitty, 1978; Candido et al., 1991; Brennan et al., 2003).

While a large rodent literature on SS processing can be found, reports often lack a standardized methodology, which makes interpretation difficult. For instance, some researchers administered SSs specifically during the acquisition phase (e.g., Bower et al., 1965) or during the extinction phase (e.g., Grossen and Bolles, 1968), or both (e.g., Dillow et al., 1972). Further, although most of the rodent studies tested female animals, evidence suggests that the existence of sex-related differences in safety processing in avoidance learning (Beck et al., 2011). Avoidance paradigms themselves also vary; some studies used lever-press discriminated avoidance (e.g., Dillow et al., 1972), free-operant avoidance (e.g., Hurwitz et al., 1972), shuttle-box avoidance (e.g., Galvani and Twitty, 1978), or jumping avoidance (e.g., Candido et al., 1991). In addition, the SS is usually a white or flashing light (e.g., Candido et al., 1991; Beck et al., 2011), but a “darkness SS” (e.g., Jacobs et al., 1983) or auditory SS (e.g., Fernando et al., 2014) has also been used. Furthermore, data on extinction learning, in which the aversive events no longer occur and the previously learned responding is expected to gradually decline, are inconsistent. While some researchers reported facilitation of extinction by the administration of SSs (Grossen and Bolles, 1968; Moscovitch and LoLordo, 1968; Weisman and Litner, 1969; Roberts et al., 1970; Jacobs et al., 1983), others found no effect (Dillow et al., 1972; Candido et al., 1991; Fernando et al., 2014). In light of the described methodological heterogeneity and inconsistent findings, translation of animal research into a clinical population is very limited. While a few attempts to test SS processing in humans have been reported (Jovanovic et al., 2005; Schiller et al., 2008; Pollak et al., 2010), all were based on classical fear conditioning, rather than operant avoidance paradigms.

The current study is the first to test the role of SSs in the acquisition and extinction of conditioned avoidance behavior in humans. We used a computer-based task that captures key features of common paradigms used to assess avoidance behavior in rodents (Sheynin et al., 2014a). On this task, which is reminiscent of a spaceship videogame, participants control a spaceship, shoot an enemy spaceship to obtain points, and hide in designated screen areas to protect against on-screen aversive events. Prior work has tested learning of SSs on this task using a conditioned discrimination procedure, where one visual signal predicted the occurrence of an aversive event, whereas another signal was associated with its non-occurrence (Molet et al., 2006; Sheynin et al., 2014a). While both prior studies demonstrated that participants successfully discriminated between on-screen stimuli and showed minimal responding during the SS, the specific contribution of the SS to avoidance behavior was not assessed. Another study that used a similar paradigm provided evidence that participants’ learning is sensitive to the visual context; manipulation of the context had a prominent effect on associative learning (Byron Nelson and del Carmen Sanjuan, 2006). Here, we extended these prior studies to test how the inclusion of visual SSs affects avoidance behavior. Importantly, the SS was a discrete on-screen cue, which was presented during the intertrial interval (ITI) and explicitly signaled a period of non-threat. Here, we refer to such cues as SSs, although there are open questions as to whether such stimuli are actually perceived and/or processed as SSs [see Beck et al. (under review)].

In addition to the effect of SSs, we were interested to investigate how individual differences affect avoidance behavior on the current paradigm. In a recent study, Beck et al. (2010) showed that female sex and behaviorally inhibited temperament (i.e., a tendency to avoid or withdraw from novel social and non-social situations), two factors associated with vulnerability to anxiety in humans (Kagan et al., 1989; Pigott, 1999), were each associated with facilitated acquisition of avoidance responding in rats. Using a computer-based task similar to the one employed in the current study, we have recently paralleled these animal findings and demonstrated that sex and inhibited temperament similarly affect avoidance behavior in humans (Sheynin et al., 2014a). Here, we expected to replicate these findings and further extend them to extinction learning. Given that animal models of anxiety vulnerability show resistance to extinction of avoidance behavior (Servatius et al., 2008), we expected anxiety-vulnerable individuals to persist with the exaggerated avoidance responding even when aversive events no longer occur. In sum, in this study, we have tested how anxiety vulnerability and SSs affect acquisition and extinction of avoidance behavior in humans. We hypothesized that both anxiety vulnerability and presence of SSs would facilitate learning of avoidance behavior. If the effect of SSs is dependent on individual differences, this could suggest a personalized approach to treat mental disorders associated with pathological avoidance.

## Materials and Methods

### Participants

Participants were 122 healthy young adults (Rutgers University-Newark undergraduate students; mean age 20.7 years, SD 3.6; 54.1% female). Participants were recruited via a departmental subject pool, in which available research studies are posted and students sign up to participate in exchange for research credits in a psychology class. Participants were randomly but evenly assigned to one of two experimental groups (*n* = 61) given different versions of the computer-based task (with or without the presence of an SS). Participants were tested individually; the participant and experimenter sat in a quiet testing area during the experiment. All participants provided written informed consent and the experiment was approved by the local research ethics committee and conducted in accordance with guidelines established by the Federal government and the Declaration of Helsinki for the protection of human participants.

### Questionnaire

All participants completed the tridimensional personality questionnaire (TPQ), a self-report questionnaire, which consists of 100 true/false items asking how the individual feels or behaves in various daily situations, and provides scores relating to three orthogonal personality dimensions (Cloninger et al., 1991). One personality dimension assessed by the TPQ, which the authors termed harm avoidance (HA), is defined as behavioral inhibition in response to novel or aversive situations (Cloninger, 1986, 1987). In line with our recent work (Sheynin et al., 2014a), and in agreement with reports from other groups (e.g., Mardaga and Hansenne, 2007; Baeken et al., 2009; Wilson et al., 2011; Bailer et al., 2013), we used this subscale to assess inhibited temperament in the current study. The other two dimensions assessed by the TPQ are reward dependence (RD), defined as marked response to rewarding stimuli, and novelty seeking (NS), defined as exploratory activity in response to novel stimulation. Based on our recent findings (Sheynin et al., 2014a), we predicted that HA scores would be related to avoidance learning in the current study, whereas RD and NS scores were not expected to show significant relationships with learning.

### Escape–avoidance task

**Figure 1:**
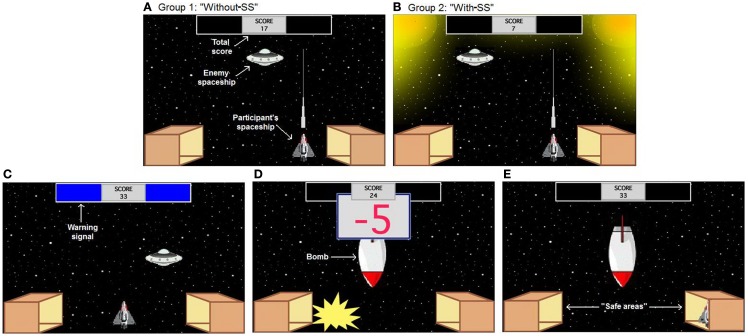
**Computer-based escape–avoidance task: one enemy spaceship appears randomly in one of six locations on the screen, approximately every 1 s**. The participant’s goal is to gain points by shooting and destroying this spaceship (1 point for each hit). **(A,B)** The experimental groups differ in the appearance of the ITI. **(A)** In the first group (without-SS), background was the same as the one during the other task periods, **(B)** whereas in the second group (with-SS), two lights were visualized at both upper corners of the screen. **(C)** The warning period includes two colored rectangles at the top of the screen, which appear every 20 s and remain visible for 5 s. **(D)** On acquisition trials, the warning period is always followed by appearance of a bomb, which remains on-screen for 5 s (bomb period). The bomb period is divided into five segments of equal duration; during each segment, there is an explosion and loss of 5 points to a maximum of 25 points. **(E)** At the bottom corners of the screen, there are two box-shaped areas representing “safe areas.” Moving the participant’s spaceship to one of those boxes is defined as “hiding.” While hiding, the participant’s spaceship can not be destroyed and no points can be lost, but neither can the participant shoot the enemy spaceship and gain points. Labels shown in white text are for illustration only and do not appear on the screen during the task.

To test escape–avoidance behavior, participants from both experimental groups were administered a computer-based task, which took the form of a spaceship videogame. The task was conducted on a Macintosh computer programed in the SuperCard language (Solutions Etcetera, Pollock Pines, CA, USA) and followed a similar design as recently described (Sheynin et al., 2014a; Figure 1). The keyboard was masked except for three keys, labeled “ ←,” “ →,” and “FIRE,” which the participants used to perform the task. In the task, participants controlled a spaceship and could move it to one of five horizontal locations at the bottom of the screen, by using the left and the right arrow keys. An enemy spaceship appeared randomly in one of six locations on the screen. Participants were instructed to gain points by using the “FIRE” key to shoot at and destroy this enemy spaceship, which appeared in a specific location for approximately 1 s unless destroyed by the participant. Every successful hit caused an explosion of the enemy spaceship and provided a reward of 1 point.

Every 20-s, two colored rectangles (the warning signal) appeared for 5 s in a designated area at the top of the screen (warning period; Figure 1C). Color of the rectangles (pink or blue) was randomly assigned, but remained constant for each participant. Each task session consisted of 24 trials. During the first 12 acquisition trials, the warning period was always followed by appearance of a bomb for another 5 s (bomb period). The bomb period was divided into five 1-s segments; during each segment, there was an explosion and a loss of 5 points (Figure 1D), to a maximum of 25 points. The bomb period was followed by a 10-s ITI during which participants could gain points without any risk of aversive events. During the subsequent 12 extinction trials, no bombs appeared and each warning period was followed by a 15-s ITI. The two experimental groups differed in the appearance of the ITI on the acquisition trials; in the first group (without-SS), the background was the same as during the other task periods (Figure 1A), while in the second group (with-SS), the background during ITI included two lights at the two upper corners of the screen (SS; Figure 1B).

Both experimental groups included two box-shaped areas representing “safe areas” at the bottom corners of the screen (Figure 1E). Moving the participant’s spaceship to either one of those boxes was defined as “hiding.” While hiding, the participant’s spaceship could not be destroyed and no points could be lost, but neither could the participant shoot the enemy spaceship and gain points. Hiding during the bomb period represented an ER and terminated point loss, while hiding during the warning period represented an avoidance behavior and could cause the complete omission of point loss; in both cases, if the participant emerged from hiding before the end of the bomb period, point loss resumed and response could not be recorded as an AR. Importantly, participants were not given any explicit instructions about the safe areas or the hiding response. At the beginning of the experiment, the participants saw the following instructions. “You are about to play a game in which you will be piloting a spaceship. You may use LEFT and RIGHT keys to move your spaceship, and press the FIRE key to fire lasers. Your goal is to score as many points as you can. The number of points will appear on the top of the screen. Good luck!” Participants were then given 1 min of practice time, during which they could shoot the enemy spaceship but no signals or bombs appeared. This practice period also included an SS in the with-SS group. Twelve trials followed, each defined by the appearance of the warning signal; the start of a new trial was not explicitly signaled to the participant. A running tally at the top of the screen showed the current points accumulated; this tally was not allowed to fall below 0, to minimize frustration among participants.

### Data analysis

Every 100 ms, the program recorded whether the participant’s spaceship was inside or outside one of the boxes. To assess avoidance behavior, percentage of time spent hiding during the 5-s warning period was recorded on each of the 12 acquisition and 12 extinction trials. In addition, following Sheynin et al. (2014a), two dependent variables were defined to describe specific aspects of avoidance: AR rate (percentage of acquisition trials on which an AR was made) and AR duration (percentage of the warning period during which the participant’s spaceship was hidden, averaged across trials). Importantly, to consider only hiding that was part of an AR, only acquisition trials where an AR was made were included in the analyses of AR duration. By definition, all ARs resulted in avoidance of any point loss on a specific trial; longer AR duration indicated that a participant made a response earlier during the warning period and remained hiding longer overall on that trial. To assess ERs, the percentage of each bomb period during which the participant’s spaceship was hidden was recorded for each acquisition trial. Finally, to analyze overall performance on the task, total points gained during the entire session, number of shooting attempts (presses on the FIRE key), and participants’ locomotion (presses on the LEFT or RIGHT keys) were recorded. Due to a computer failure, number of shooting attempts and locomotion data for one participant were not recorded.

To compare the two experimental groups (with-SS versus without-SS), we used *t*-test for continuous values and chi-square for categorical values, with Yates continuity correction for 2 × 2 tables. To test association of sex, personality, and presence of SS with the escape–avoidance behavior, we used stepwise linear regressions. Predictor variables were sex, score on the TPQ subscales (NS, HA, and RD), and experimental group. Dependent variables were average hiding during the warning period on acquisition and extinction trials, and average hiding during the bomb period on acquisition trials. Similar analyses were also conducted on AR rate, AR duration, and the different task performance variables (total points, shooting, and locomotion). Internal consistency of the different questionnaire subscales was analyzed using Cronbach’s α with reverse scoring for individual questions taken into account. Statistical analyses were conducted using SPSS version 17.0 (SPSS Inc., Chicago, IL, USA). Alpha was set to 0.050, effects that did not approach significance (*p* > 0.100) were not reported.

## Results

On the NS, HA, and RD subscales of the TPQ questionnaire, mean (SD) values were 16.8 (4.8), 12.8 (7.8), and 18.4 (4.3), respectively. For the 34, 34, and 30 questions comprising NS, HA, and RD subscales, inter-item reliability was 0.689, 0.900, and 0.678, respectively. No correlations were found between TPQ subscales (Pearson correlations, all *p* ≥ 0.600). Participants assigned to the two experimental groups did not differ on sex, age, or any of the TPQ subscale scores (all *p* > 0.100).

On the computer task, one participant gained only one point during the entire session (more than 2.5 SD from group mean) and demonstrated extremely high locomotive activity (more than 8 SD from group mean); data from this participant were excluded from all the behavioral analyses reported below.

**Figure 2:**
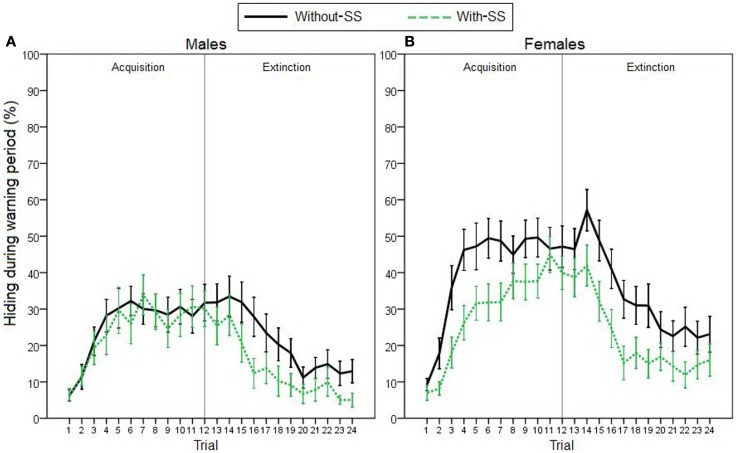
**Acquisition and extinction of avoidance behavior, represented by percentage of time spent hiding during the warning period in (A) male participants with and without SS (*n* = 26 and 29, respectively), and in (B) female participants with and without SS (*n* = 34 and 32, respectively)**. On the acquisition phase, sex was the best predictor of performance (stepwise linear regression, *p* = 0.007). *Post hoc* regression analyses separately for each sex revealed that females’ hiding could be predicted by the experimental group (with-SS versus without-SS; *p* = 0.023). On the extinction phase, performance was best predicted by level of acquisition responding and experimental group (*p* < 0.001). Vertical gray lines represent the end of the acquisition phase. Error bars indicate SEM.

For the remaining participants, to assess avoidance behavior on the task, we first analyzed percentage of time spent hiding during the 5-s warning period (Figure 2). On the acquisition phase, stepwise linear regression revealed that hiding could be predicted by a model including sex as the only predictor variable [*R*^2^ = 0.059, *R* = 0.244, *F*(1,119) = 7.524, *p* = 0.007]; females acquired avoidance behavior faster and to a higher degree than males. Further, due to an apparent effect of experimental group on females’ acquisition learning (Figure 2B), we performed *post hoc* regression analyses, separately for each sex (predictor variables were TPQ subscales and experimental group). As hypothesized, analyses revealed that females’ hiding could be predicted by experimental group [*R*^2^ = 0.078, *R* = 0.280, *F*(1,64) = 5.438, *p* = 0.023]; females in the “with-SS” group acquired slower than those in the “without-SS” group. In males, however, similar analysis identified no variables as significant predictors (all *p* > 0.300).

On the extinction phase, hiding could be predicted by sex [*R*^2^ = 0.064, *R* = 0.253, *F*(1,119) = 8.123, *p* = 0.005], as well as by both sex and experimental group [*R*^2^ = 0.128, *R* = 0.358, *F*(2,118) = 8.647, *p* < 0.001]. Adding experimental group to the model accounted for significant additional variance (*p* = 0.004); males and participants in the “with-SS” group extinguished faster than females and those in the “without-SS” group. Crucially, since participants’ hiding on the extinction phase was positively correlated with their earlier hiding during the acquisition phase (Pearson correlation, *r* = 0.655, *p* < 0.001), we used a hierarchical multiple regression to repeat the latter analysis while controlling for behavior during that phase. First, average hiding on the acquisition phase was entered as predictor variable. As expected, hiding during extinction could be predicted solely by amount of hiding during acquisition [*R^2^* = 0.429, *R* = 0.655, *F*(1,119) = 89.434, *p* < 0.001]. On the next step of the analysis, a stepwise linear regression was used to test whether any of the other variables (sex, TPQ subscales, and experimental group) could account for significant additional variance in hiding during extinction, beyond that accounted for by hiding during acquisition. When experimental group was added as a predictor variable, the model could account for significant additional variance (*p* = 0.045); therefore, extinction behavior could be best predicted by a model including both acquisition hiding and the presence of SS [*R*^2^ = 0.448, *R* = 0.670, *F*(2,118) = 47.954, *p < *0.001]. Participants in the “with-SS” group, who showed less avoidance acquisition, demonstrated faster extinction learning than their counterparts.

**Figure 3:**
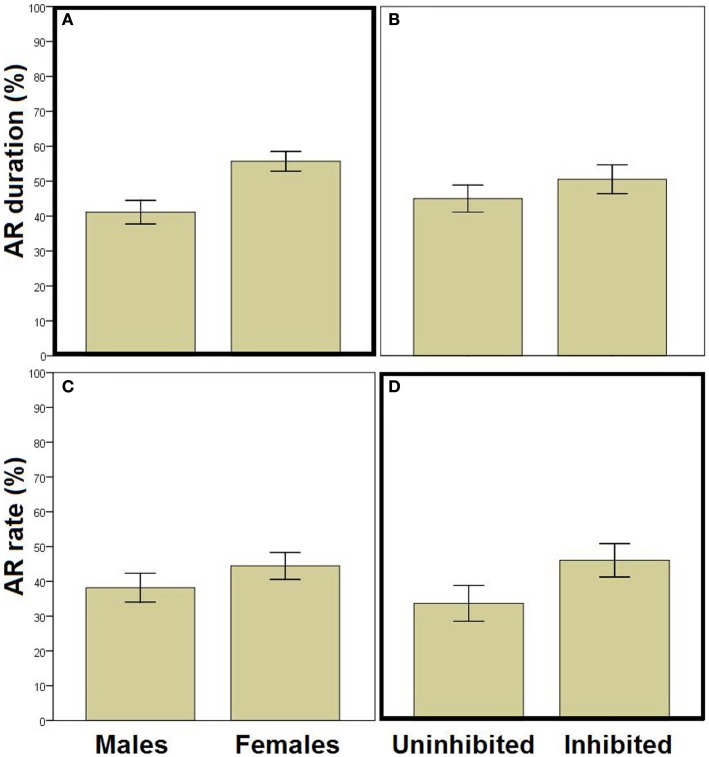
**Assessment of specific aspects of avoidance**. **(A,B)** AR duration (percentage of warning period spent hiding, for those trials where an AR was made) in males versus females [**(A)** *n* = 42 and 54, respectively] and in inhibited versus uninhibited participants [**(B)** participants scoring in the upper and lower thirds on HA; *n* = 37 and 26, respectively]; AR duration could be predicted by a model including sex as the only predicting variable (stepwise linear regression, *p* = 0.001). **(C,D)** AR rate (percent of trials with an AR) in males versus females [**(C)** *n* = 55 and 66, respectively] and in inhibited versus uninhibited participants [**(D)** *n* = 43 and 36, respectively]; AR rate was higher in the inhibited than in the uninhibited participants. However, the relationship between AR rate and HA did not reach statistical significance (all *p* > 0.200). Error bars indicate SEM.

**Figure 4:**
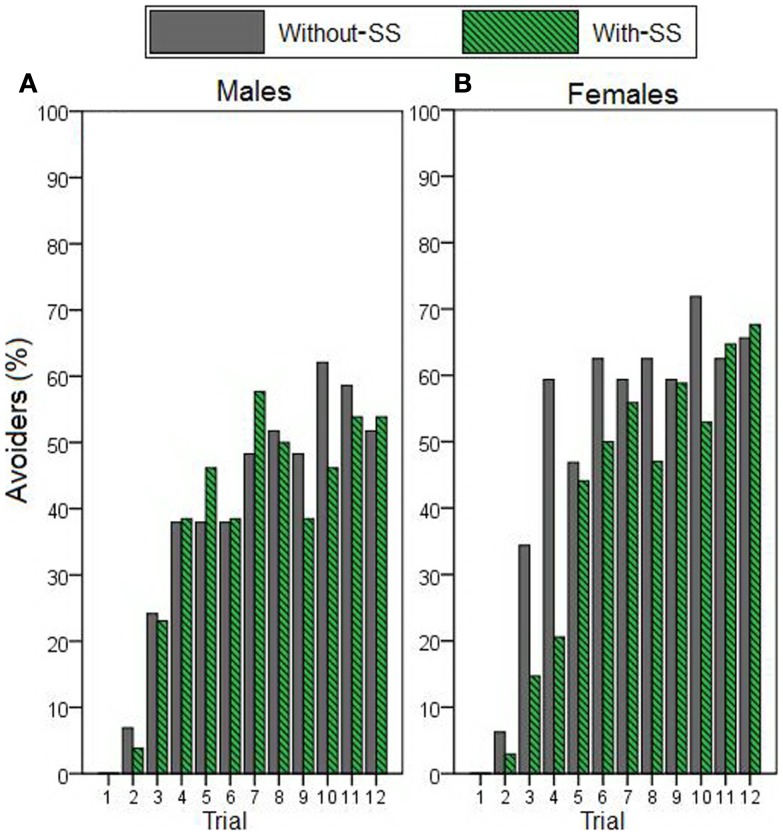
**Avoidance response rate across acquisition trials – represents the percentage of participants who exhibited an AR on each trial (avoiders)**. Data describe **(A)** male participants with and without SS (*n* = 26 and 29, respectively), and **(B)** female participants with and without SS (*n* = 34 and 32, respectively). A main stepwise linear regression, as well as *post hoc* regressions separately for each sex, identified no variables as significant predictors (all *p* > 0.100).

We next used stepwise linear regression to examine two specific aspects of AR (Figure 3). AR duration (calculated only on trials where an AR was made) could be predicted by a model including sex as the only predictor variable [*R*^2^ = 0.105, *R* = 0.323, *F*(1,94) = 10.985, *p* = 0.001; Figures 3A,B]; females demonstrated longer duration of hiding during the warning period. Considering AR rate (Figures 3C,D), highly inhibited individuals (i.e., those scoring in the top third of HA scores) demonstrated more ARs than their uninhibited counterparts (i.e., those scoring in the lower third of HA scores). However, stepwise linear regression indicated that neither HA nor any of the other potential predictor variables accounted for significant variability in AR rate (all *p* > 0.200). Interestingly, when AR rate data were displayed separately for each trial (as the percentage of “avoiders,” i.e., participants exhibiting an AR on that trial; Figure 4), on some trials the “with-SS” group included numerically fewer female avoiders than the “without-SS” group (Figure 4B). However, *post hoc* regression analyses separately for each sex (predictor variables were TPQ subscales and experimental group) identified no variables as significant predictors (all *p* > 0.100).

**Figure 5:**
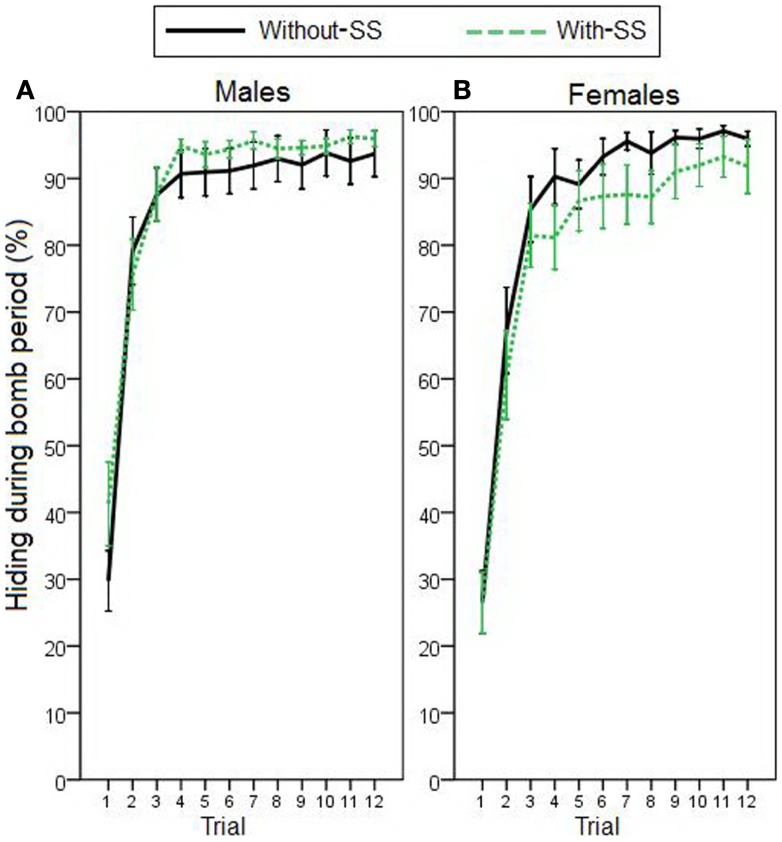
**Acquisition of ER, represented by percentage of hiding during the bomb period in (A) male participants with and without SS (*n* = 26 and 29, respectively), and in (B) female participants with and without SS (*n* = 34 and 32, respectively)**. Stepwise linear regression indicated that neither sex, experimental group, nor personality scores could significantly predict escape behavior (all *p* > 0.100). Error bars indicate SEM.

Then, we assessed ER on the task by analyzing hiding during the 5-s bomb period on the acquisition phase (Figure 5). Stepwise linear regression indicated that no predictor variables could significantly predict variance in ER, meaning that there were no significant effects of sex, personality, or experimental group (all *p* > 0.100).

**Figure 6:**
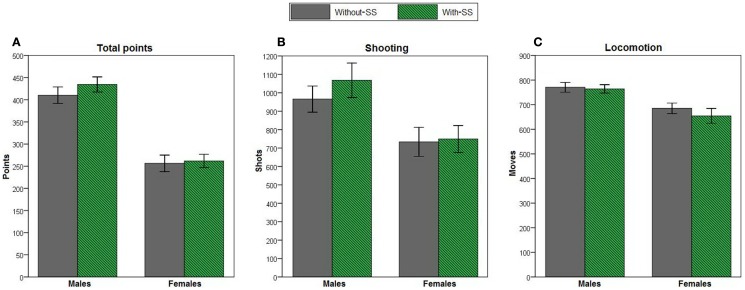
**Total points, total shooting attempts (presses on the FIRE key), and locomotion (presses on the LEFT or RIGHT keys) on the computer-based task in male and female participants with-SS (*n* = 26 and 34, respectively) and without-SS (*n* = 29 and 32, respectively)**. Due to a computer failure, shooting and locomotion data for one participant were not recorded. For all three performance measures, scores could be predicted by a model that included sex as the only predictor variable (all *p* ≤ 0.001); **(A)** females earned fewer points, **(B)** made fewer attempts to shoot, and **(C)** showed less locomotion than males. Error bars indicate SEM.

Lastly, we assessed overall task performance (Figure 6). Stepwise linear regression revealed that a model that included sex as the only predictor variable could be used to predict total points [*R*^2^ = 0.423, *R* = 0.650, *F*(1,119) = 87.271, *p* < 0.001], shooting [*R*^2^ = 0.092, *R* = 0.304, *F*(1,118) = 12.023, *p* = 0.001], and locomotion [*R*^2^ = 0.127, *R* = 0.356, *F*(1,118) = 17.113, *p* < 0.001]. Across the entire task session, females earned fewer points (Figure 6A), made fewer attempts to shoot (i.e., fewer FIRE key presses; Figure 6B), and showed less locomotion (i.e., fewer LEFT and RIGHT key presses; Figure 6C) than males. Adding experimental group and personality variables into the models did not account for additional variance (all *p* > 0.100).

## Discussion

The purpose of the current study was to examine the effects of an SS on avoidance acquisition and extinction in humans. Participants were tested on a computer-based escape–avoidance task meant to capture several key features of avoidance paradigms commonly used in rodents. Here, participants were divided into two experimental groups that differed in whether an SS was presented during acquisition. Results showed that the presence of an SS during the acquisition phase of the task impaired acquisition (in females) and facilitated extinction of the learned avoidance behavior (Figure 2). Results also generally replicated our prior findings with this task (Sheynin et al., 2014a); specifically, females demonstrated longer duration of hiding on trials where an AR was made (AR duration; Figure 3A), and participants with inhibited temperament showed a higher AR rate than uninhibited participants (Figure 3D), although the latter relationship fell short of statistical significance in the current study. These findings, and limitations of the current study, are discussed further below.

### Safety signals and acquisition of avoidance

Prior studies in rodents had demonstrated that the presence of an SS during acquisition facilitated acquisition of the AR (e.g., Bower et al., 1965; Dillow et al., 1972; Hurwitz et al., 1972; Candido et al., 1991). However, in the current study, presence of an SS did not affect (in males) or even impaired (in females) acquisition of avoidance. One possible explanation for this discrepancy is that, in the rat studies, the SS is usually contingent on (and appears immediately after) a successful AR or ER; as such, it may provide positive reinforcement for the behavioral response (Bolles and Grossen, 1969; Dillow et al., 1972). In contrast, the SS in the current study was non-contingent, and appeared at the end of each bomb period, whether or not an AR or ER was made. In fact, when non-contingent SSs are used in rodent studies, facilitated acquisition is not observed (Fernando et al., 2013). An interesting avenue for future work may be to compare the effect of contingent versus non-contingent SS on avoidance acquisition in humans. Moreover, this work suggests sex-related differential effect of the SS; it is possible that the SS caused higher relaxation in females (Denny and Weisman, 1964), which was generalized to the warning period and resulted in reduced avoidance. Such a differential effect of an SS in males and females is in agreement with the differential utilization of SSs by male and female rodents (Beck et al., 2011), and emphasizes the need to include both sexes in future animal and human research.

### Safety signals and extinction of avoidance

In contrast to the sex-dependent effect of SSs on acquisition in the current task, there was a main effect of SSs on extinction. Specifically, participants for whom the SS was present during the ITI on acquisition trials subsequently extinguished avoidance faster than those with no SS. This finding is consistent with several reports showing that inclusion of SSs in the acquisition phase of free-operant avoidance facilitates extinction (Roberts et al., 1970; Jacobs et al., 1983; Beck et al., 2011; Fernando et al., 2014). One explanation for this effect is that there is a context shift between acquisition, where SS is present during the ITI, and extinction, where it is not (Roberts et al., 1970). Consistent with this explanation, rodent studies that administered the SS during both acquisition and extinction failed to show any effect of SSs on extinction (Dillow et al., 1972; Candido et al., 1991).

### Sex differences

Females in the current study showed more avoidance behavior, regardless of the presence of an SS. This is consistent with previous reports, which showed that females acquire avoidance behavior faster than their male counterparts, in both rodents (Beatty and Beatty, 1970; Van Oyen et al., 1981; Beck et al., 2010) and humans (McLean and Hope, 2010; Sheynin et al., 2014a). As in our prior study (Sheynin et al., 2014a), the current study also found a specific association between sex and AR duration, with females hiding longer than males during the warning signal on trials where an AR was demonstrated. The current study further showed that females continued to demonstrate more avoidance behavior even on extinction trials when the aversive events no longer occurred.

An important feature of the current paradigm is the motivational conflict between the option to avoid the aversive event by hiding in one of the safe areas versus the option to gain points by staying in the central area and shooting the enemy spaceship. It is possible that the observed sex differences are the result of distinct sensitivities to these appetitive and aversive components of the task. This idea is consistent with recent work, which suggested that female and male rats process reward and punishment differently on a decision-making task (van den Bos et al., 2012). It is also possible that males in the current study had greater reward sensitivity, which caused them to delay the AR and remain in the open to accrue points, until the last possible moment before the bomb arrived (Li et al., 2007). This idea of higher reward-seeking in males is supported by the fact that males scored more total points than females (Figure 6A), and made more attempts to obtain such reward (i.e., increased shooting rate; Figure 6B), but did not differ from females on responding during the bomb period, when no reward was available (i.e., ERs; Figure 5).

In the current task, increased hiding during the warning period typically means that the participant entered the safe area soon after the onset of the warning signal, and remained there throughout the remainder of the warning period and the subsequent bomb period. Thus, increased duration of hiding during the warning signal did not serve to better avoid the upcoming point loss, but rather prevented the participant from obtaining further reward (points). This pattern is in line with recent findings by van den Bos et al. (2012), who showed that female rats demonstrated a disadvantageous strategy on a decision-making paradigm, which resulted in less reward (fewer sugar pellets) than obtained by male rats. This non-optimal behavior might be related to the pathological avoidance behavior demonstrated by anxious individuals, and might represent a behavioral risk factor that underlies females’ vulnerability to develop anxiety disorders (Pigott, 1999).

Interestingly, we also found that females gained fewer points overall, as well as making fewer attempts to gain these points, indexed as number of shooting attempts (Figures 6A,B). This could represent a decreased reward sensitivity in females (Li et al., 2007), but it might also be the case that females were simply less experienced or less motivated at playing videogames (Pfister, 2011). While it seems reasonable to believe that most college-age participants had at least some prior exposure to computer games, future studies should specifically address and control for this variable. In the current study, it appears unlikely that male–female differences simply reflected differences in experience with computer games, since AR duration assesses the timing of a learned response, rather than the response itself. In fact, both genders executed ARs at the same rate (Figure 3C). In addition, following a report that showed that exaggerated locomotor activity can mask avoidance differences in rodents (Aguilar et al., 1998), we analyzed locomotion in the current study. However, females’ exaggerated avoidance on the current task can not be simply attributed to increased locomotor activity making them more likely to enter the hiding areas, since females actually showed less locomotor activity than males (Figure 6C).

### Inhibited temperament differences

Participants who reported an inhibited temperament in the current study (as assessed by the HA subscale of the TPQ questionnaire) tended to show more ARs than uninhibited participants. This is consistent with our recent report, where AR rate could be reliably predicted by inhibited temperament on a similar computer-based task (Sheynin et al., 2014a). Interestingly, this relationship did not reach statistical significance in the current study. While this could merely represent minor differences across participant samples, it could also be the result of subtle variations in the task design (e.g., presence of a “control-signal” in the earlier study versus the SS in the current study). Future studies should follow up on this finding and further investigate the exact nature of this relationship.

### Implications for therapy

The overall effect of SSs on avoidance behavior in the current study has potential therapeutic relevance. Anxiety disorders, as well as post-traumatic stress disorder, are characterized by impaired extinction learning, reflected in patients’ tendency to keep emitting ARs, even when the aversive outcomes no longer occur (Graham and Milad, 2011). An attempt to promote extinction is often made in cognitive–behavioral therapies via exposure techniques, where individuals are exposed to the feared stimulus or outcome in the absence of actual threat (Balooch et al., 2012). The current study, in which extinction was facilitated by the presence of an SS during a prior acquisition phase, suggests that individuals might benefit from the exposure to non-threat cues during or near the time of the traumatic experience. Importantly, given the slower extinction learning exhibited by female participants in the current study, females might benefit more from the use of SSs. Further, future work should test whether a similar positive effect on extinction learning can be obtained if SSs are administered during the extinction phase itself. Indeed, it was argued that therapeutic procedures that improve patients’ general sense of safety and security would reduce avoidance in agoraphobic patients (Rachman, 1984; Sartory et al., 1989).

It is also important to note that most of the research on processing of SSs in humans is based on classical fear conditioning rather than avoidance learning, mainly because of the dearth of adequate tools to investigate conditioned avoidance in humans. The current study investigated a purely cognitive form of avoidance learning that involved a point loss in a computer game. While the current and previous studies suggested that such paradigms are sufficient for triggering more avoidance behavior in individuals with anxiety vulnerabilities (Sheynin et al., 2013, 2014a), a direct comparison of cognitive versus fear-evoked avoidance should be a focus of future work. In addition, future work could include screening for drug use, to control for its possible involvement in the reported behavioral differences (Sheynin et al., 2014b). Lastly, future work should consider adapting the current task to further promote the study of behavioral differences in anxious individuals. For instance, manipulating the Pavlovian contingency between the warning signal and the aversive event (e.g., compare probabilistic versus deterministic designs) or the instrumental contingency of the hiding response [e.g., manipulate the frequency of the protective outcome (AR)] might add uncertainty to the task, and thus, better dissociate individual differences (McEvoy and Mahoney, 2012). Moreover, adapting the current task for acquisition across multiple sessions would allow the study of the “warm-up” phenomenon, where the subject starts a training session at a lower performance level than what was performed at the end of the previous training session. Since a lack of warm-up is exhibited by the inhibited WKY rat strain (Servatius et al., 2008), we hypothesize that inhibited human subjects might show a similar impairment.

### Active versus passive avoidance

It is important to discuss the type of avoidance behavior that is addressed by the current computer-based task. In the current task, the hiding response protects the participant from the aversive event (AR); participants who enter the safe area soon after onset of the warning signal typically have longer AR duration than those who remain in the central area until right before the bomb appears. AR duration is therefore roughly comparable to response latency in rodent active avoidance tasks, where a rat can emit a lever-press or other response (AR) after onset of the warning signal but before arrival of the aversive shock. However, although AR in the current task is clearly an active behavioral strategy that requires the initial move of the participant’s spaceship from the central to a safe area, it also includes an important passive property. By definition, both AR rate and AR duration require a persistent hiding state, where the initial active response (entering the safe area) is followed by a passive response (staying in the safe area through the rest of the warning period and through the entire bomb period to completely avoid any explosion and any point loss). Thus, in this task participants can learn a unique avoidance behavior that includes both active and passive properties, both of which have been demonstrated to be abnormal in rodents with increased anxiety levels (Dubrovina and Tomilenko, 2007; Beck et al., 2010).

This idea of a mixed avoidance pattern has been investigated in humans; for example, adolescent running away behavior may reflect passive avoidance in males but both passive and active avoidance in females (De Man et al., 1994). Clinically, agoraphobia may be associated with strong passive avoidance but weak active avoidance (Zinbarg et al., 1992). These studies suggest that inhibited temperament and female sex might be differentially associated with active and passive avoidance. In future, the current computer-based task could be adapted to specifically target these types of behavior, and potentially, analyze them separately within each tested individual. Specifically, active avoidance could be assessed by a single key press that would terminate/prevent the aversive event (parallel to the rat lever-press response), whereas passive avoidance would be the requirement to withdraw from the shooting response (Arcediano et al., 1996).

## Conclusion

This is the first study to examine how non-contingent SSs affect acquisition and extinction of escape–avoidance behavior in male and female humans. In this study, we found that administering such signals during the acquisition phase specifically attenuated avoidance behavior, without affecting other behavioral measures such as acquisition of ERs or overall performance on the computer-based task. As the participants in the current study were healthy young adults, our findings shed light on specific vulnerability factors that confer risk to develop anxiety disorders in future, and also suggest how a better understanding of SSs may promote therapeutic approaches in individuals who develop pathological avoidance.

## Author Contributions

All authors participated in the design of the project. Jony Sheynin conducted the data analysis and prepared the initial draft of the manuscript. All authors contributed to revising the manuscript, approved the final version, and agreed to be accountable for all aspects of this work.

## Conflict of Interest Statement

The authors declare that the research was conducted in the absence of any commercial or financial relationships that could be construed as a potential conflict of interest.
